# Developmental delay in motor skill acquisition in Niemann-Pick C1 mice reveals abnormal cerebellar morphogenesis

**DOI:** 10.1186/s40478-016-0370-z

**Published:** 2016-09-01

**Authors:** Paola Caporali, Francesco Bruno, Giampiero Palladino, Jessica Dragotto, Laura Petrosini, Franco Mangia, Robert P. Erickson, Sonia Canterini, Maria Teresa Fiorenza

**Affiliations:** 1 Department of Psychology, Section of Neuroscience and “Daniel Bovet” Neurobiology Research Center, Sapienza University of Rome, Via dei Sardi 70, 00185 Rome, Italy; 2 IRCCS Fondazione Santa Lucia, Via del Fosso di Fiorano 64, 00179 Rome, Italy; 3 Department of Pediatrics, University of Arizona, 1501 N Campbell Ave, Tucson, AZ 85724-5073 USA

**Keywords:** Lysosomal storage disorders, Cholesterol, Cerebellar cortex development, Motor behavior, 2-hydroxypropyl-β-cyclodextrin, Dysmyelination

## Abstract

Niemann-Pick type C1 (NPC1) disease is a lysosomal storage disorder caused by defective intracellular trafficking of exogenous cholesterol. Purkinje cell (PC) degeneration is the main sign of cerebellar dysfunction in both NPC1 patients and animal models. It has been recently shown that a significant decrease in Sonic hedgehog (Shh) expression reduces the proliferative potential of granule neuron precursors in the developing cerebellum of *Npc1*^*−/−*^ mice. Pursuing the hypothesis that this developmental defect translates into functional impairments, we have assayed Npc1-deficient pups belonging to the milder mutant mouse strain *Npc1*^*nmf164*^ for sensorimotor development from postnatal day (PN) 3 to PN21. *Npc1*^*nmf164*^*/ Npc1*^*nmf164*^ pups displayed a 2.5-day delay in the acquisition of complex motor abilities compared to *wild-type* (*wt*) littermates, in agreement with the significant disorganization of cerebellar cortex cytoarchitecture observed between PN11 and PN15. Compared to *wt*, *Npc1*^*nmf164*^ homozygous mice exhibited a poorer morphological differentiation of Bergmann glia (BG), as indicated by thicker radial shafts and less elaborate reticular pattern of lateral processes. Also BG functional development was defective, as indicated by the significant reduction in GLAST and Glutamine synthetase expression. A reduced VGluT2 and GAD65 expression also indicated an overall derangement of the glutamatergic/GABAergic stimulation that PCs receive by climbing/parallel fibers and basket/stellate cells, respectively. Lastly, Npc1-deficiency also affected oligodendrocyte differentiation as indicated by the strong reduction of myelin basic protein. Two sequential 2-hydroxypropyl-β-cyclodextrin administrations at PN4 and PN7 counteract these defects, partially preventing functional impairment of BG and fully restoring the normal patterns of glutamatergic/GABAergic stimulation to PCs.

These findings indicate that in *Npc1*^*nmf164*^ homozygous mice the derangement of synaptic connectivity and dysmyelination during cerebellar morphogenesis largely anticipate motor deficits that are typically observed during adulthood.

**Electronic supplementary material:**

The online version of this article (doi:10.1186/s40478-016-0370-z) contains supplementary material, which is available to authorized users.

## Introduction

Niemann-Pick type C (NPC) is an inherited lysosomal storage disorder, ultimately fatal and presenting with variable neurovisceral symptoms, age of onset and life span [1]. In spite of broad clinical features, impaired fine motor skills, unsteady gait and balance deficits are the earliest sign of neurological manifestation [2]. The most recent incidence estimate is 1.12 affected patients per 100,000 live births, although this value is likely underestimated because of misdiagnosis [3]. The defect is due to mutations in the genes *NPC1* (95 % of cases) or *NPC2*, encoding for proteins that cooperatively mediate the egress from endosomes/lysosomes of exogenous cholesterol brought to the cells by the low density lipoprotein (LDL)/clathrin-coated pit pathway [4]. The role of NPC1/NPC2 proteins is particularly important in neural cells because cholesterol does not cross the blood–brain barrier once it is fully established after birth [5], making the adult brain mostly dependent on endogenously-derived cholesterol. Accordingly, cholesterol *de novo* synthesis occurs in both neurons and astrocytes during early postnatal neurogenesis, thereafter becoming most prominent in astrocytes [6].

Progressive Purkinje cell (PC) degeneration [1, 7] leading to ataxia, represents the most important neuropathological feature of the disease, although the reason for the selective vulnerability of this neuronal population is currently unknown. Because patients do not apparently show early developmental defects and also because most neuropathological signs appear in *Npc1*^−/−^ mice in the juvenile/young adult age, the possibility that early cerebellum development processes are impaired by NPC1-deficiency has mostly been neglected. However, the development and functional maturation of mouse cerebellar cortex is a long-lasting process encompassing the first three postnatal weeks [8], during which the need for cholesterol is likely to maximize to face the intense glial/neuronal cell proliferation and migration, neurite outgrowth, synaptogenesis and myelin formation. These findings may explain why *Npc1* loss-of-function affects the cerebellum more severely compared to other brain regions such as the hippocampus and cortex, whose development is largely completed prior to birth [9, 10]. It has been recently shown that, due to premature exit from the cell cycle, there are a decreased number of granule neurons (GNs) and a 20–25 % reduction in cerebellar lobule size at the end of cerebellar development [11]. This leads to a deficiency of GNs in the Inner Granular Layer (IGL), which may contribute to the later PC degeneration. In line with the robust mitogenic activity Shh exerts on GNs [12], *Shh* mRNA levels were found to be significantly reduced at the time of final divisions of GN precursors [11]. Besides GNs, also Bergmann glia (BG) responds to Shh [13] by differentiating in relationship with PC migration, dendritogenesis, synaptogenesis and maturation [14], suggesting that Npc1-deficiency also affects the normal pattern of BG differentiation.

Among the animal models of NPC disease, the *Npc1*^*nmf164*^ mouse is of particular interest because it harbors a single nucleotide substitution (A to G at cDNA bp 3163) causing an aspartate-to-glycine substitution (D1005G) in the cysteine-rich luminal loop, conferring to the NPC1 protein a partial loss of activity as observed in most common human mutations [15]. By assessing the physical and sensorimotor development of pre-weaning *Npc1*^*nmf164*^ homozygous mice, we have observed a significant delay in the acquisition of complex motor skills compared to *wt* littermates, which likely indicates an impairment of the cerebellar circuitry functionality. Therefore, we hypothesized that the differentiation of glial cells, including BG and oligodendrocytes, as well as the expression/localization patterns of functional markers of glutamatergic and GABAergic transmission might be altered in *Npc1*^*nmf164*^ homozygous mice. The evidence we provide in this study, showing that cerebellar morphogenesis is significantly damaged in *Npc1*^*nmf164*^ homozygous mice substantially confirms our hypothesis.

2-Hydroxypropyl-β-cyclodextrin, a drug promoting cholesterol movement from late endosomes to the metabolically active pool of cholesterol in the cytosol [16], has been shown to slow the appearance of ataxic symptoms in NPC1 disease mouse [17, 18] and cat models [19], representing the major treatment currently studied in NPC1 patients. In light of this evidence we assessed whether the administration of this drug rescued the abnormal cerebellar morphogenesis of *Npc1*^*nmf164*^ mice.

## Materials and methods

### Animals and treatments

*Npc1*^*nmf1*64/*nmf164*^ mice with BALB/cJ background (hereafter named *Npc1*^*nmf1*64^ mice) obtained from heterozygous crosses were exposed to a 12 h light–dark cycle, receiving food and water *ad libitum*. The genotypes of pups were identified by PCR analysis of tail DNA as described [15]. Because a preliminary evaluation ruled out any gender effect on preweaning and adult behavioral performances, male and female mice were grouped together for analyses. Preweaning and adult behavioral performances were analyzed on the same cohorts of 10 *Npc1*^*nmf164*^ and 10 *wt* littermates, obtained from 5 litters made of at least 7 pups. Treatment with 2-hydroxypropyl-β-cyclodextrin (hereafter named CD; average degree of substitution of 0.67 of hydroxypropyl groups per glucose unit, MW ~1369 Da, catalog number H-107, Sigma Aldrich, Milan, Italy) was performed by two subsequent subcutaneous injection of either a 20 % solution (w/v; 4000 mg/Kg body weight) of CD in PBS, or plain PBS (sham, control) to 4- and 7-day-old mice *Npc1*^*nmf164*^ and *wt* littermates [11, 20]. The effect of CD administration on behavioral performances of preweaning pups was assessed on a cohort of 10 *Npc1*^*nmf164*^ and 10 *wt* littermates (5 pups either PBS- or CD-injected/genotype), obtained from 5 litters made of at least 7 pups.

**Fig. 1:**
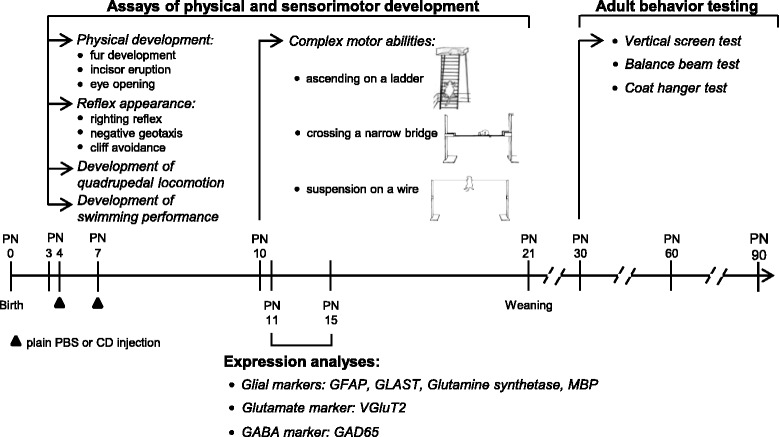
Experimental design. A schematic summary of behavioral assessment and expression analyses of glial and neuronal cell markers of *Npc1* ^*nmf164*^ and age-matched *wt* mice. PN: postnatal day; CD: 2-hydroxypropyl-β-cyclodextrin

A scheme summarizing the time schedule of behavioral assays and expression pattern analyses is reported in Fig. 1. Experimental protocols and related procedures were approved by the Italian Ministry of Public Health. All efforts were made to minimize animal suffering, according to European Directive 2010/63/EU.

### Preweaning behavior assessment

From postnatal day (PN) 3 to PN21, pups were separated from their dams daily between 9:00 a.m. and 3:00 p.m. for a maximum of 15 min, and tested for physical, postural, locomotor and complex motor behavior development in a warmed environment (30–32 °C) [21–23]. Behavioral assessment evaluated the development of physical parameters (body weight, eye opening, fur appearance, incisor eruption), locomotion (pivoting, crawling, quadrupedal locomotion), swimming performance (direction and limb use), reflex appearance (surface righting reflex, negative geotaxis, cliff avoidance) and complex motor behaviors (ascending a ladder, crossing a narrow bridge, suspension on a wire). Besides direct behavioral observations, videos were also recorded throughout the entire test cycle. To avoid the possibility of order effect(s), the test sequence was administered to each pup in random order for each test. The attribution of the dominant behavior to a specific category in each observation period was made blindly with regard to pup’s genotype. Categorization was considered reliable only when judgments were consistent (inter-rate reliability > 0.9). The test batteries used for the assessment of physical and sensorimotor development were as follows:

- *Physical development*. The *body weight* was measured daily in the interval PN3-PN21 and *eye opening*, *fur appearance* and *incisor eruption* were evaluated by visual inspection.
- *Development of quadrupedal locomotion*. Fluent forward movements with all limbs supporting the whole body and the pelvis elevated were analyzed from PN3 to PN15 by using Ethovision XT software (Noldus, The Netherlands). The pup was placed on a board and video-recorded for 120 s to analyze the following locomotion categories: (i) *pivoting*, turning movements by broad swipes with forepaws, using only one hindlimb as a pivot and having the pelvis anchored to the ground; (ii) *crawling*, dragging the body forward or pushing it backward by undulating movements of the trunk and often dragging the hindlimbs in an extended position with foot soles facing upward; (iii) *quadrupedal locomotion,* smooth and coordinated walking, in which the body is supported in sequence by different numbers of feet in combination, suitable for variegated velocities and without any directional bias. The developmental acquisition of the various locomotion categories was determined as dominant behavior according to the rating scale of Table 1.

**Table 1:**
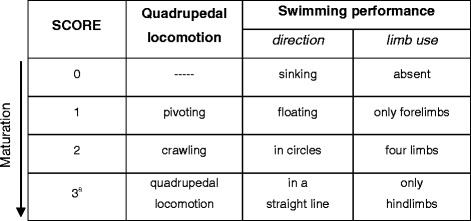
Rating scale of the development of quadrupedal locomotion and swimming performance

^a^the highest score corresponds to the fully-developed behavior
- *Development of swimming performance.* The pup was gently released in a glass tank (cm 100 × 50 × 20) filled with warmed (35 °C) water and allowed to swim freely. The parameters *swimming direction* and *limb use* were evaluated and scored according to the rating scale of Table 1.
- *Reflex appearance*. (i) *Surface righting reflex*: the pup was placed gently on its back and the time to turn over on the belly was recorded (allotted time 30 s). (ii) *Negative geotaxis*: the pup was placed on an inclined (30°) plane with the head pointing downwards and the time to face up to the slope was recorded (allotted time 60 s). (iii) *Cliff avoidance*: the pup was placed on an edge with forepaws and nose just over the edge and the time to retract itself by backward and/or sideward movements was recorded (allotted time 60 s).
- *Development of complex motor behaviors*. Because the acquisition of complex motor abilities requires the complete maturation of basic reflexes such as the grasping response, which normally appears by the end of the first postnatal week [22], the development of complex motor behaviors was scored from PN10 on. (i) *Ascending a ladder*: the pup was placed on a steel ladder (cm 15 × 25, 20 rungs, 1 cm apart, inclination angle 25°) with top leaning against a platform holding littermates. The ability to ascend the ladder within 120 s was evaluated and the day of the first successful performance was recorded. (ii) *Crossing a narrow bridge*: the pup was placed on the start platform connected by a plywood bridge (40 × 1 × 3 cm) to the goal platform holding littermates. The ability to traverse the bridge within 120 s was evaluated and the day of the first successful crossing was recorded. (iii) *Suspension on a wire*: the pup was suspended by its forepaws on a wire (2 mm diameter and 50 cm long) extended horizontally between two poles (30 cm high). The suspension time and the first suspension with the 4 limbs (hind limb suspension) were recorded (allotted time 60 s).

### Adult behavior assessment

PN30, PN60 and PN90 *Npc1*^*nmf164*^ and *wt* littermates were subjected to two daily sessions (morning and afternoon) of the following consecutively administered tests assessing motor behavior [24]: (i) *Vertical screen:* the mouse was placed on a horizontal wire screen (cm 15x25, wire diameter 2 mm, spaced at 1 cm). The screen was rapidly turned to vertical position with the mouse facing the floor at the lower edge. The latency to turn upward and to climb to the upper edge was measured during 60 s. This test was performed as the first one of the morning session and was not repeated in the afternoon of that day. (ii) *Balance beam:* the mouse was placed perpendicularly at the center of a horizontal round beam (covered with paper tape, outer diameter 2 cm, length 1 m, divided into 10 sections and placed 50 cm above a padded surface). The retention time and the number of beam sections crossed during 180 s were recorded and the results of morning and afternoon trials were averaged. (iii) *Coat hanger:* the mouse was suspended in the middle of the horizontal bar of a coat hanger (diameter 3 mm, length 35 cm, placed 30 cm above a padded surface) with its forepaws. The body position of the animal was observed for 60 s and scored as follows: 0, a fall within 10 s; 1, grasping the hanger with one limb; 2, grasping the hanger with two limbs; 3, grasping the hanger with three limbs; 4, grasping the hanger with four limbs; 5, actively escaping to the end of the bar. The values of morning and afternoon trials were averaged.

These tests were selected because they were similar to those we had exploited in behavioral analyses of preweaning pups in terms of functions evaluated and experimental setting.

### Western blot assays

For Western blot analyses, total proteins of PN11 and PN15 *Npc1*^*nmf164*^ and *wt* littermate cerebella (4 mice/genotype/age) were extracted with RIPA buffer (Sigma Aldrich) supplemented with protease and phosphatase inhibitors (Roche Life Science, Indianopolis, IN, USA). The protein concentration was routinely determined by Bradford’s colorimetric assay (Bio-Rad, Milan, IT). Equal amounts of total protein/lane were fractionated by electrophoresis on a 4–12 % gradient SDS-polyacrylamide gel (Bolt® Bis-Tris Plus gels, Life Technologies, Carlsbad, CA, USA) or 10 % gel pre-cast (Bio-Rad). Fractionated proteins were transferred to PVDF membranes (GE Healthcare, Little Chalfont, UK) and then processed for Western blot analyses. When proteins of interest had very different electrophoretic migrations, such as in the case of glutamine synthetase and MBP, membranes were cut into strips to be probed with different antibodies. The primary and secondary antibodies used are reported in Table 2. To evaluate the effect of CD administration on protein levels, similar assays were also performed on PN15 *wt* and *Npc1*^*nmf164*^, either sham- or CD-treated (4 mice/genotype/treatment) mice.

**Table 2:** Antibodies used

Antibody		Company	Dilution	
			WB^a^	IHC^a^
Primary	Anti-GFAP	Santa Cruz Biotechnolgy, Santa Cruz, CA, USA; #sc-33673	1:500	1:50
	Anti-EAAT1 or GLAST	AbCam, Cambridge, UK; #ab416	1:1500	1:250
	Anti-glutamine synthetase	AbCam; #ab73593	1:2000	1:333
	Anti-VGluT2	Thermo Fisher Scientific, Rockford, IL; USA; #PA5-25653	1:1000	1:50
	Anti-GAD65	AbCam; #ab26113	1:2000	1:200
	Anti-MBP	Sigma-Aldrich Inc., St. Louis, MO, USA; #M3821	1:500	1:100
	Anti-β-actin	AbCam; #ab6276	1:1000	-----
Secondary	Horseradish peroxidase-conjugated goat anti-rabbit IgG	Thermo Fisher Scientific; #32460	1:200	-----
	Horseradish peroxidase-conjugated goat anti-mouse IgG	Thermo Fisher Scientific; #32430	1:650	-----
	Horseradish peroxidase-conjugated goat anti-mouse IgG2a	Santa Cruz Biotechnolgy; #sc-2061	1:3000	-----
	Biotinylated goat anti-rabbit IgG	Vector Laboratories, Burlingame, CA; #PK-6101	-----	1:200
	Biotinyted goat anti-mouse IgG	Vector Laboratories; #PK-6102	-----	1:200

^a^ *WB* Western blot assay, *IHC* immunohistochemistry

### Immunohistochemistry

PN15 *Npc1*^*nmf164*^ and *wt* littermates (4 mice/genotype) were deeply anaesthetized by intraperitoneal injection of a mixture of xylazine (20 mg/Kg) and ketamine (34 mg/Kg) and then transcardially perfused with 4 % PFA in 0.1 M PBS. Brains were removed and post-fixed overnight at 4 °C in 4 % PFA. For MBP detection, PFA-fixed brains were dehydrated, embedded in Paraplast Tissue Embedding Medium (Leica Biosystems, Milan, Italy) and serially sectioned (slice thickness 8 μm). Sagittal sections were mounted on X-tra Adhesive glass slides (Leica Biosystems), de-waxed with xylene, rehydrated and washed in PBS. The detection of other glial and neuronal cell markers was performed on cryosections. To this end, fixed brains were cryoprotected with sucrose (30 %, w/v, in PBS), embedded in FSC22 Clear R Frozen Section Compound (Leica Biosystems) and serially sectioned (slice thickness 8 μm) using a Leica CM 1900 cryostat. For GLAST detection, cryosections were subjected to 20 min fixation in acetone (−20 °C), which significantly improved antigen detection [25]. Paraffin sections and cryosections were then processed for epitope unmasking and endogenous peroxidases inactivation. For antigen unmasking, sections were incubated (5 min × 2) in 10 mM sodium citrate, pH 6.0 in a microwave oven and then in 0.3 % H_2_O_2_ for 15 min at RT to inactivate endogenous peroxidases. A 2 h incubation in a blocking solution made of 0.5 % BSA in PBS preceded the incubation of sections with anti-GLAST and anti-MBP antibodies. For the detection of VGlut2, GFAP, Glutamine synthetase and GAD65 the blocking solution was supplemented with 0.1 % Triton X-100. The incubation of sections with primary antibodies lasted approximately 18 h at 4 °C and was followed by several washes in PBS before exposure to the appropriate secondary antibody (see Table 2 for details). Antibody-antigen complexes were revealed with Vectastain Elite ABC Kit (Vector Laboratories Inc., Burlingame, CA, USA) followed by DAB Peroxidase Substrate Kit (Vector Laboratories Inc.), according to manufacturer’s instructions. Immunodetection specificity was assessed by omitting the primary antibody. Images were obtained using a Zeiss Axioplan microscope equipped with a Sony nex-3 N mirror-less camera (Sony Europe Limited, Milano, Italy) and processed using ImageJ NIH software (National Institutes of Health, Bethesda, MD).

VGluT2- and GAD65-positive puncta were quantitated in 3–4 sagittal sections of 4 mice/genotype as previously described [26, 27], with slight modifications. Images were acquired using a Zeiss Axioplan microscope at 100X magnification (Neofluar, 0.7–1.30) and a Sony nex-3 N mirror-less camera. For each antibody, at least 8 image fields of lobule II and lobule X were acquired along the molecular layer starting from the pial surface. The abundance of VGluT2- and GAD65-positive puncta was determined in regions of interest (ROI) of 6500 μm^2^ and 3200 μm^2^, respectively, randomly selected in outer and inner molecular layers by the “cell counter” function of ImageJ NIH software. The number of GAD65-positive puncta around the PC’s soma was also determined. Only VGluT2- and GAD65-positive puncta having a high-to-moderate staining and a diameter of 0.3–1.3 μm were counted. All determinations were performed blindly and independently by two investigators. Because no significant difference was observed between counts of lobule II and lobule X microscopic fields of *wt* or *Npc1*^*nmf164*^ mice, data were pooled.

### Statistical analyses

Statistical analyses were performed with STATISTICA 8 (StatSoft) software. Data were first tested for normality (Wilk-Shapiro’s test) and homoscedasticity (Levene’s test), and then analyzed by unpaired two-tailed Student’s *t* test or two-way ANOVAs for independent (genotype, treatment) and repeated (age) measures, followed by Bonferroni’s *post-hoc* test. When data did not fully meet parametric assumptions or were ordinal (locomotion and swimming measures), comparisons between groups were performed by Mann-Whitney’s *U* test. To control for alpha inflation, i.e. the proportion of type I errors among all rejected null hypotheses, the False Discovery Rate (FDR) was set to 0.05 and estimated through a bootstrap procedure [28]. Differences were considered to be significant at the *p* ≤ 0.01 level.

## Results

### *Npc1*^*nmf164*^ mice show a delay in the acquisition of complex motor skills requiring fine motor coordination and balance

**Fig. 2:**
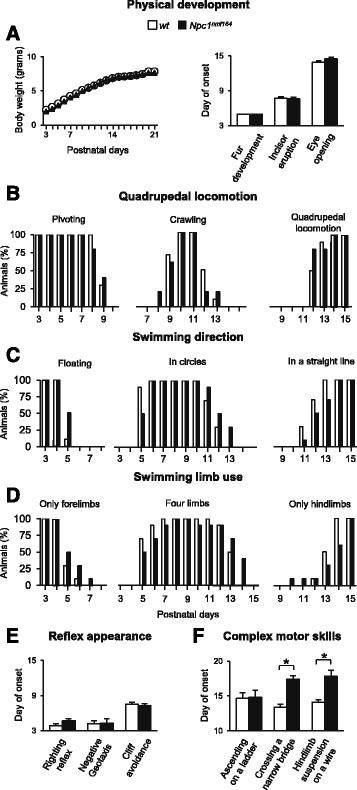
*Npc1* ^*nmf164*^ pups show a delay in the acquisition of complex motor skills requiring fine motor coordination and balance. **a** Line graph indicates body weight values of experimental group mice of increasing age. Histograms indicate the day of onset of physical development landmarks. **b**-**d** Histograms indicate the fraction of: pups engaged in pivoting, crawling or quadrupedal locomotion (**b**); pups floating, swimming in circles or in a straight line (**c**); pups paddling with only forelimbs, four limbs or only hindlimbs (**d**) in the PN3-PN15 time interval. **e**-**f** Histograms indicate the day of onset of sensorimotor reflexes (**e**) and complex motor skills (**f**). Note that Npc1-deficiency delays the acquisition of complex motor behaviors requiring fine motor coordination and balance, whereas it does not influence physical and sensorimotor development. **a**, **e**, **f** Data are expressed as mean ± SEM. **b**-**d** Data are expressed as percentages of animals displaying the behavior. * *p* ≤ 0.01

Because sensorimotor reflexes and motor skills normally appear with a definite timing during the first 3 weeks after birth, they represent a useful tool to assess early postnatal neural development [29]. We therefore evaluated the acquisition of several developmental milestones in the physical and sensorimotor development of *Npc1*^*nmf164*^ mice from PN3 until weaning (PN21). Body weight, fur appearance, incisor eruption and eye opening were recorded as indexes of physical growth and development, observing no difference between *Npc1*^*nmf164*^ and *wt* littermates (Fig. 2a). All pups similarly increased their body weight in the interval PN3-PN21 (main effect of genotype: *F*_1,18_ = 0.80, *p* = 0.78; main effect of age: *F*_18,324_ = 364.14, *p* < 0.00001; interaction between genotype and age: *F*_18,324_ = 0.47, *p* = 0.97), and showed dorsal and ventral fur appearance after PN5 (main effect of genotype: *Z* = −1.30, *p* = 0.47), incisor eruption after PN7 (main effect of genotype: *Z* = 0.32, *p* = 0.80), and eye opening after PN14 (main effect of genotype: *Z* = −1.58, *p* = 0.14). To analyze the locomotor development we determined the appearance of the dominant locomotory categories *pivoting* (turning with circular motions), c*rawling* (moving forward/pushing backward the body) and *quadrupedal locomotion* (showing fluent and swift forward movements), observing no difference between *Npc1*^*nmf164*^ and *wt* littermates (Fig. 2b, Table 3). Namely, pups showed pivoting from PN3 to PN9, crawling at PN10-11 and quadrupedal locomotion since PN12. We also determined the development of swimming abilities and observed no effect of genotype: all pups floated with asynchronous limb movements at PN4, swam in circles at PN5, swam in a straight line at PN12 and displayed the adult swimming pattern (paddling only the hindlimbs) after PN14 (Fig. 2c-d, Table 3). We then recorded the appearance of reflexes as surface righting reflex, negative geotaxis and cliff avoidance, which involve vestibular, tactile and proprioceptive systems [30]. Negative geotaxis and cliff avoidance are more representative of sensory ability, whereas the surface righting reflex is more representative of motor ability [22]. *Npc1*^*nmf164*^ mice displayed a timing of reflex appearance that matched that of *wt* littermates (Fig. 2e), exhibiting similar appearance of surface righting reflex (main effect of genotype: *Z* = −1.70, *p* = 0.10) and negative geotaxis since PN4 (main effect of genotype: *Z* = 0.38, *p* = 0.74), as well as cliff avoidance since PN7 (main effect of genotype: *Z* = 0.20, *p* = 0.85).

**Table 3:** Statistical analysis outputs of quadrupedal locomotion and swimming performance development in *Npc1* ^*nmf164*^ and *wt* littermates^a^

Age^b^	Quadrupedal locomotion	Swimming performance	
		Direction	Limb usage
PN3	*Z* = 1.00; *p* = 0.74	*Z* = 0.00; *p* = 1.00	*Z* = 0.00; *p* = 1.00
PN4	*Z* = −1.00; *p* = 0.74	*Z* = 0.00; *p* = 1.00	*Z* = 0.00; *p* = 1.00
PN5	*Z* = 1.00; *p* = 0.74	*Z* = 1.90; *p* = 0.14	*Z* = 0.89; *p* = 0.48
PN6	*Z* = −1.00; *p* = 0.74	*Z* = 0.00; *p* = 1.00	*Z* = 1.09; *p* = 0.48
PN7	*Z* = −0.61; *p* = 0.74	*Z* = 0.00; *p* = 1.00	*Z* = 1.00; *p* = 0.74
PN8	*Z* = 0.97; *p* = 0.53	*Z* = 0.00; *p* = 1.00	*Z* = 0.00; *p* = 1.00
PN9	*Z* = −0.59; *p* = 0.63	*Z* = 0.00; *p* = 1.00	*Z* = 0.00; *p* = 1.00
PN10	*Z* = 0.00; *p* = 1.00	*Z* = 0.00; *p* = 1.00	*Z* = −1.00; *p* = 0.74
PN11	*Z* = −1.09; *p* = 0.48	*Z* = −1.09; *p* = 0.48	*Z* = −1.00; *p* = 0.74
PN12	*Z* = 1.37; *p* = 0.28	*Z* = 0.89; *p* = 0.48	*Z* = 0.00; *p* = 1.03
PN13	*Z* = −1.13; *p* = 0.44	*Z* = 1.83; *p* = 0.28	*Z* = 0.89; *p* = 0.48
PN14	*Z* = −1.45; *p* = 0.48	*Z* = 0.00; *p* = 1.00	*Z* = 2.18; *p* = 0.14
PN15	*Z* = 0.00; *p* = 1.00	*Z* = 0.00; *p* = 1.00	*Z* = 1.00; *p* = 0.74

^a^Experimental groups were compared at increasing postnatal days by Mann–Whitney *U* test

^b^ *PN* postnatal day

In the mouse, complex motor abilities requiring fine limb coordination, balance and muscle strength are normally acquired by the end of the second postnatal week. Three tasks (*ascending a ladder*, *crossing a narrow bridge* and *suspension on a wire*) allowed us to differentiate the contribution of motor coordination and balance from that of grip and muscle strength. *Npc1*^*nmf164*^ pups acquired these abilities with a significant delay compared to *wt* littermates (Fig. 2f). Indeed, whereas *wt* pups crossed the narrow bridge in its entire length and hanged on the wire with four limbs after PN14, *Npc1*^*nmf164*^ mice crossed the bridge only at PN17 (main effect of genotype: *Z* = −2.54, *p* = 0.01) and developed the four-limb hanging ability at PN18 (main effect of genotype: *Z* = −2.98, *p* = 0.004). In contrast, grip ability and muscle strength developed similarly in *Npc1*^*nmf164*^ and *wt* littermates, as shown by their similar ability to ascend the ladder after PN15 (main effect of genotype: *Z* = 0.27, *p* = 0.80) and to hang on the wire for a longer time with increasing age (main effect of genotype: *F*_1,18_ = 1.09, *p* = 0.31; main effect of age: *F*_10,180_ = 3.23, *p* = 0.0008; interaction between genotype and age: *F*_10,180_ = 0.20, *p* = 0.99).

The possibility of evaluating the efficacy of CD to rescue the developmental delay in motor skills acquisition of *Npc1*^*nmf164*^ and *wt* littermates was hampered by the hyperactivity of mouse pups elicited by the injection *per se*. Both CD-treated and sham group pups, regardless of genotype resisted our attempts to perform motor behavior assessments.

### Motor deficits of *Npc1*^*nmf164*^ mice become more severe in adulthood

To fully characterize motor phenotype in adults, PN30, PN60 and PN90 *Npc1*^*nmf164*^ and *wt* littermates were subjected to a battery of tests including *Vertical screen*, *Balance beam*, and *Coat hanger*.

**Fig. 3:**
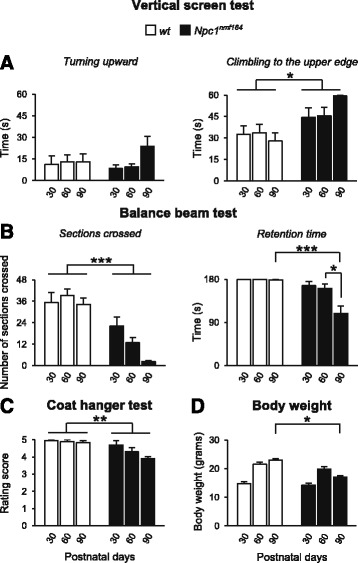
*Npc1* ^*nmf164*^ adult mice display motor deficits after PN30. **a**-**d** Histograms indicate: latency values to turn upward and climb to the upper edge in the Vertical screen test (**a**); number of sections crossed and retention time values in the Balance beam test (**b**); rating score values in the Coat hanger test (**c**); body weight values (**d**) of experimental group mice of increasing age. All data are expressed as mean ± SEM. * *p* < 0.01, ** *p* < 0.001, *** *p* < 0.0001

The *Vertical screen* test (similar to the *ascending on a ladder*) investigates the climbing response that requires good grip and muscle strength (Fig. 3a). In this test *Npc1*^*nmf164*^ mice reached the upper edge of the screen more slowly than *wt* littermates, even if both genotypes turned upwards with similar time (*turning upward:* main effect of genotype: *F*_1,18_ = 0.12, *p* = 0.73; main effect of age: *F*_2,36_ = 1.91, *p* = 0.16; interaction between genotype and age: *F*_2,36_ = 1.52, *P* = 0.23); (*climbing to the upper edge:* main effect of genotype: *F*_1,18_ = 11.31, *p* = 0.004; main effect of age: *F*_2,36_ = 0.59, *p* = 0.57; interaction between genotype and age: *F*_*2*,36_ = 2.63, *p* = 0.09).

The *Balance beam* test (similar to *crossing a narrow bridge*) measures fine motor coordination and balance (Fig. 3b). When placed on an elevated round beam, *Npc1*^*nmf164*^ mice crossed significantly fewer beam sections than *wt* mice did and significantly fewer sections as days went by (main effect of genotype: *F*_1,18_ = 34.92, *p* = 0.00001; main effect of age: *F*_2,36_ = 5.08, *p* = 0.01; interaction between genotype and age: *F*_2,36_ = 4.09, *p* = 0.03). Moreover *Npc1*^*nmf164*^ mice did not differ from *wt* until PN90 in terms of retention time (main effect of genotype: *F*_1,18_ = 54.28, *p* < 0.00001; main effect of age: *F*_2,36_ = 6.48, *p* = 0.004; interaction between genotype and age: *F*_2,36_ = 6.01, *p* = 0.006).

The *Coat hanger* test (similar to *suspending on a wire*) further characterizes motor coordination by providing an “agility score” (Fig. 3c). *Npc1*^*nmf164*^ mice obtained scores lower than those of *wt* mice when suspended on the coat hanger. In fact, while *wt* mice rapidly escaped to the bar end, *Npc1*^*nmf164*^ mice did not progress to the end of the bar although they were able to grasp the bar with four limbs (main effect of genotype: *F*_1,18_ = 18.81, *p* = 0.0004; main effect of age: *F*_2,36_ = 3.80, *p* = 0.03; interaction between genotype and age: *F*_2,36_ = 2.30, *p* = 0.11).

The possibility that body weight influenced motor behavior was routinely checked before all behavioral evaluations (Fig. 3d). Body weight of *Npc1*^*nmf164*^ and *wt* mice did not differ at PN30 and PN60, while it significantly decreased in PN90 *Npc1*^*nmf164*^ mice, as previously described [15] (main effect of genotype: *F*_1,18_ = 13.35, *p* = 0.002; main effect of age: *F*_2,36_ = 125.40, *p* < 0.00001; interaction between genotype and age: *F*_2,36_ = 22.26, *p* < 0.00001).

### Bergmann glia morphogenesis and functions are defective in *Npc1*^*nmf164*^ mice

Our analysis of the gross morphology of PN15 *Npc1*^*nmf164*^ mouse cerebellum showed that the number of GNs forming the external granule layer was significantly reduced compared to age-matched *wt* mice (Additional file 1 and Additional file 2: Figure S1A-B), suggesting a defective proliferation of GN precursors similar to that previously observed in *Npc1*^*−/−*^ mice [11]. The quantification of cells incorporating BrdU (Additional file 2: Figure S1C-D) confirmed this possibility and prompted us to further analyze the cerebellar morphogenesis of these mice.

**Fig. 4:**
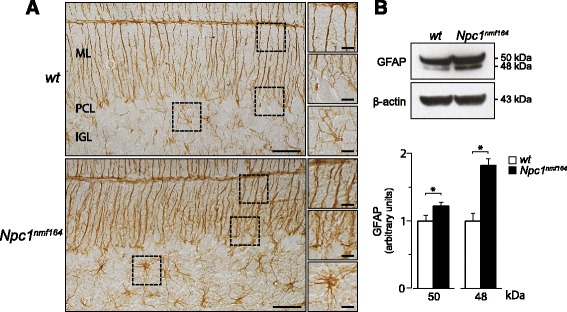
Bergmann glia morphogenesis is defective in *Npc1* ^*nmf164*^ mice. **a** Immunostaining with antibodies directed to GFAP (brown) shows that BG of PN15 *Npc1* ^*nmf164*^ mice have radial shafts that are enlarged and irregular in caliber, as well as hypertrophic astrocytes within the IGL, compared to *wt* littermates. Representative fields of parasagittal sections of *wt* and *Npc1* ^*nmf164*^ mouse cerebella are shown in the Fig.; scale bars: 50 μm. Higher magnification fields are shown on the right; scale bars: 25 μm. ML: Molecular Layer; PCL: Purkinje Cell Layer; IGL: Internal Granular Layer. **b** Western blot analysis of GFAP protein expression in cerebella of PN15 *wt* and *Npc1* ^*nmf164*^ mice. Histograms indicate the abundance (mean ± SEM) of each GFAP isoforms determined by densitometry of protein bands obtained in at least 3 independent experiments taking β-actin as internal reference. * *p* ≤ 0.01

During the first week of postnatal development, BG radial shafts span the entire molecular layer, providing the scaffold for GN migration [31] and directing the distal growth of the PC dendritic tree [32]. Further BG development favors PC dendritic arborization and synapse formation, leading to the complex reticular meshwork of the adult cerebellar cortex [14]. To determine whether Npc1-deficiency affected BG morphology and/or functional differentiation, we assessed the expression and localization pattern of glial fibrillary acidic protein (GFAP), glutamate transporter (GLAST) and Glutamine synthetase by immunohistochemistry and Western blot analysis. BG morphology was thus assessed by immunostaining histological sections of PN11 and PN15 *Npc1*^*nmf164*^ and *wt* cerebella with antibodies directed to GFAP. While no significant difference was found between *Npc1*^*nmf164*^ and *wt* mice at PN11 (Additional file 3: Figure S2), BG of PN15 *Npc1*^*nmf164*^ mice had radial shafts, which were enlarged and irregular in caliber and displayed hypertrophic astrocytes in the internal granule layer (IGL) (Fig. 4a). The overall increase in size of BG and astrocytes of *Npc1*^*nmf164*^ mice was accompanied by an abnormal increase in GFAP expression, as quantified by Western blot analysis (Fig. 4b). It is worth noting the presence of two GFAP protein bands having an apparent MW of 50 and 48 kDa, respectively, both more abundant in *Npc1*^*nmf164*^ mice compared to *wt* littermates (main effect of genotype: 48 kDa, t_6_ = 4.34, *p* = 0.005; 50 kDa, t_6_ = 3.44, *p* = 0.01). The 48 kDa protein band is generated by calpain I proteolitic cleavage [33] and increases during neurodegenerative processes [34].

**Fig. 5:**
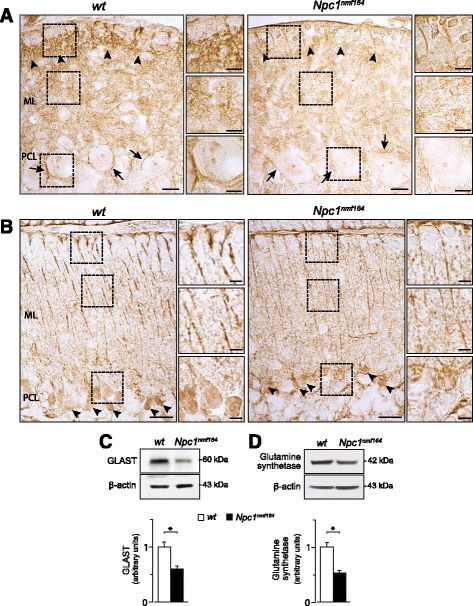
Bergmann glia function appears to be defective in *Npc1* ^*nmf164*^ mice. **a** Immunostaining with antibodies directed to GLAST (brown) shows that PN15 *Npc1* ^*nmf164*^ mice display a reduced expression of GLAST at the level of BG processes in the outer part of molecular layer (arrowheads) and around Purkinje cell soma (arrows) compared to *wt* littermates. Representative fields of parasagittal sections of *wt* and *Npc1* ^*nmf164*^ mouse cerebella are shown in the Fig.; scale bars: 10 μm. Higher magnification fields are shown on the right; scale bars: 5 μm. **b** Immunostaining with antibodies directed to Glutamine synthetase (brown) shows that PN15 *Npc1* ^*nmf164*^ mice display a reduced expression of Glutamine synthetase at the level of BG soma (arrowheads) and processes compared to *wt* littermates. Representative fields of parasagittal sections of *wt* and *Npc1* ^*nmf164*^ mouse cerebella are shown in the Fig.; scale bars: 20 μm. Higher magnification fields are shown on the right; scale bars: 5 μm. ML: Molecular Layer; PCL: Purkinje Cell Layer. **c**-**d** Western blot analyses of GLAST (**c**) and Glutamine synthetase (**d**) protein expression in cerebella of PN15 *wt* and *Npc1* ^*nmf164*^ mice. Histograms indicate GLAST (**c**) and Glutamine synthetase (**d**) abundance (mean ± SEM) determined by densitometry of protein bands obtained in at least 3 independent experiments taking the β-actin as internal reference. * *p* < 0.01

BG is normally provided with a large amount of GLAST, which is particularly abundant in the cell body and perisynaptic membranes, here preventing glutamate spillover between adjacent PCs [35]. We determined GLAST expression by immunostaining and Western blot analyses, observing a significant GLAST reduction in *Npc1*^*nmf164*^ compared to *wt* littermates (main effect of genotype: t_6_ = 4.27, *p* = 0.005) (Fig. 5a, c). Such GLAST reduction was particularly evident around PC soma, which are normally enwrapped by lamellar processes arising from BG cell bodies [8, 14, 36] and in the distal BG radial shaft close to the pial surface. *Npc1*^*nmf164*^ cerebella also displayed a significant decrease in Glutamine synthetase expression, as evaluated by both immunohistochemistry and Western blot analyses (main effect of genotype: t_6_ = 4.79, *p* = 0.003) (Fig. 5b, d). The decrease in Glutamine synthetase was stronger at the level of BG soma and milder along BG radial shafts. In spite of the abnormal morphological/functional development of BG processes, the number and localization of BG soma around PC cell bodies were apparently normal (Additional file 4: Figure S3).

### Purkinje cells of *Npc1*^*nmf164*^ mice display a reduced number of glutamatergic and GABAergic inputs

**Fig. 6:**
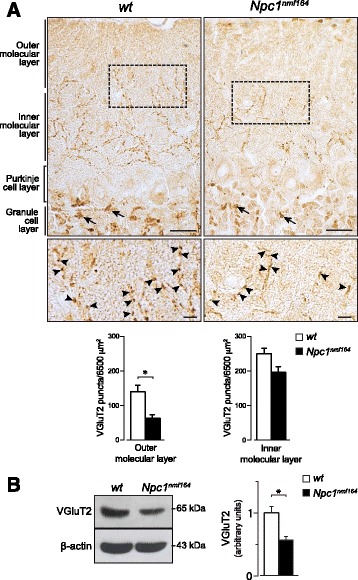
Purkinje cells of *Npc1* ^*nmf164*^ mice display a reduced number of glutamatergic inputs. **a** Immunostaining with antibodies directed to VGluT2 (brown) shows that PN15 *Npc1* ^*nmf164*^ mice display a reduced expression of VGluT2 in the outer part of molecular layer compared to *wt* littermates. Representative fields of parasagittal sections of lobule II of *wt* and *Npc1* ^*nmf164*^ mouse cerebella are shown. Upper panels: arrows indicate VGluT2-positive synapses of internal granule layer glomeruli; scale bars: 20 μm. Bottom panels: higher magnifications of selected areas. Arrowheads indicate typical VGluT2 positive puncta; scale bars: 5 μm. Histograms indicate VGluT2-positive puncta densities in the outer and inner molecular layers (mean ± SEM). **b** Western blot analysis of VGluT2 protein expression in cerebella of PN15 *wt* and *Npc1* ^*nmf164*^ mice. Histograms indicate VGluT2 abundance (mean ± SEM) determined by densitometry of protein bands obtained in at least 3 independent experiments taking the β-actin as internal reference. * *p* < 0.01

PCs display distinct anatomical and physiological compartments, which receive at least two excitatory and two inhibitory inputs on different proximal and distal sub-compartments of cerebellar cortex [36] respectively, dividing the molecular layer into outer and inner parts. In fact, thin distal PC dendrite branchlets receive glutamatergic inputs from parallel fibers and GABAergic inputs from stellate interneurons [37], whereas the thick proximal PC dendritic shafts receive synapses mostly from GABAergic basket interneurons and glutamatergic climbing fibers [38]. We studied PC glutamatergic and GABAergic inputs to PCs by immunostaining histological sections of PN15 *Npc1*^*nmf164*^ and *wt* cerebella with antibodies directed to vesicular glutamate transporter subtype 2 (VGluT2, labeling glutamatergic terminals) and glutamic acid decarboxylase 65 (GAD65, labeling GABAergic terminals). Compared to *wt* littermates, the molecular layer of *Npc1*^*nmf164*^ mouse cerebella displayed a reduced number of VGluT2-positive puncta, which was particularly pronounced at the level of outer part of molecular layer (main effect of genotype: t_6_ = 3.87, *p* = 0.008), whereas differences at the level of inner molecular layer didn’t reach statistical significance (main effect of genotype: t_6_ = 2.55, *p =* 0.04) (Fig. 6a). As expected, VGluT2 immunostaining was also detected at the level of glomeruli, where glutamatergic afferent mossy fibers synapse with granule neuron dendrites, with similar expression patterns in *wt* and *Npc1*^*nmf164*^ mice. Finally, Western blot analysis revealed a significant reduction of VGluT2 protein levels in the cerebellum of PN15 *Npc1*^*nmf164*^ mice (main effect of genotype: t_6_ = 4.75, *p* = 0.003) (Fig. 6b).

**Fig. 7:**
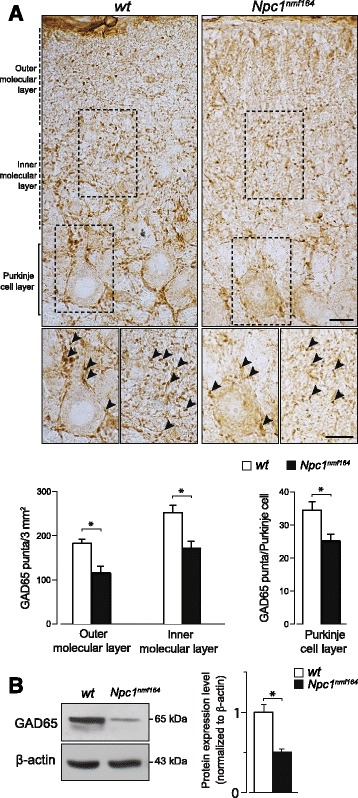
Purkinje cells of *Npc1* ^*nmf164*^ mice display a reduced number of GABAergic inputs. **a** Immunostaining with antibodies directed to GAD65 (brown) shows a reduced density of GAD65-positive puncta (arrowheads) around Purkinje cell soma and throughout the entire molecular layer of PN15 *Npc1* ^*nmf164*^ mice compared to *wt* littermates. Representative fields of parasagittal sections of lobule II *wt* and *Npc1* ^*nmf164*^ mice cerebella are shown in the Fig.; scale bars: 10 μm. Higher magnifications are shown in bottom panel insets; Arrowheads indicate typical GAD65-positive puncta; scale bars: 10 μm. Histograms indicate the density of GAD65-positive puncta in outer and inner molecular layers and Purkinje cell layer. **b** Western blot analysis of GAD65 protein expression in cerebella of PN15 *wt* and *Npc1* ^*nmf164*^ mice. Histograms indicate GAD65 abundance (mean ± SEM) determined by densitometry of protein bands obtained in at least 3 independent experiments taking the β-actin as internal reference. * *p* = 0.01

The analysis of GAD65 expression patterns also showed that GABAergic inputs were significantly reduced in *Npc1*^*nmf164*^ cerebella. To investigate this issue, we arbitrarily divided the molecular layer into outer, inner and PC layers and determined the density of GAD65-positive puncta in each layer, observing a significant reduction of puncta in molecular and PC layers of *Npc1*^*nmf164*^ vs *wt* mice (main effect of genotype: outer molecular layer: t_6_ = 3.64, *p* = 0.01; inner molecular layer: t_6_ = 3.44, *p* = 0.01; Purkinje cell layer: t_6_ = 3.58, *p* = 0.01) (Fig. 7a). Reduced GAD65 expression was also confirmed by Western blot analysis (main effect of genotype: t_6_ = 3.71, *p* = 0.01) (Fig. 7b). In spite of the reduced abundance of GAD65-positive puncta, the number and localization of GABAergic interneurons along the molecular layer appeared similar in *Npc1*^*nmf164*^ and *wt* mice, as determined by hematoxylin/eosin Y staining and parvalbumin immunostaining (Additional file 4: Figure S3).

### *Npc1*^*nmf164*^ mice display defective myelin maturation

**Fig. 8:**
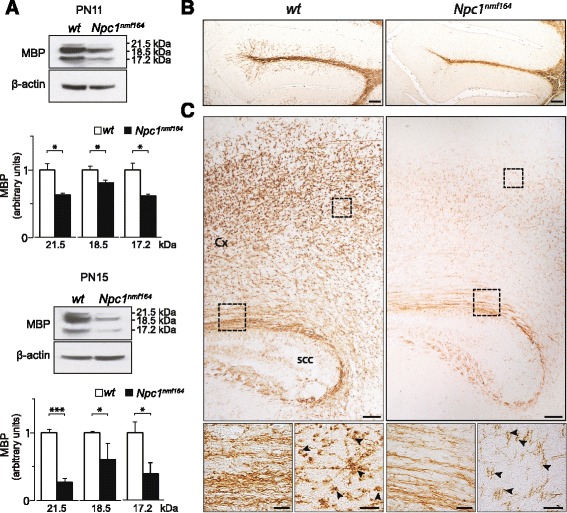
Oligodendrocyte maturation is impaired in *Npc1* ^*nmf164*^ mice. **a** Western blot analysis of MBP protein expression in cerebella of PN11 and PN15 *wt* and *Npc1* ^*nmf164*^ mice. Histograms indicate the abundance (mean ± SEM) of each isoform determined by densitometry of protein bands obtained in at least 3 independent experiments taking β-actin as internal reference. * *p* ≤ 0.01, *** *p* < 0.0001. **b** Immunostaining with antibodies directed to MBP (brown) shows that PN15 *Npc1* ^*nmf164*^ mouse cerebella display a reduction of MBP expression at the level of PC axons and white matter compared to *wt* littermates. Representative fields of parasagittal sections of lobule III of PN15 *wt* and *Npc1* ^*nmf164*^ mouse cerebella are shown in the Fig.; scale bars: 100 μm. **c** Immunostaining with antibodies directed to MBP (brown) showing that PN15 *Npc1* ^*nmf164*^ mice display a poorer oligodendrocyte differentiation as indicated by the reduced length of MBP-positive processes that typically radiate from oligodendrocyte soma (arrowheads), compared to *wt* littermates. Representative fields of parasagittal sections of PN15 *wt* and *Npc1* ^*nmf164*^ mouse cerebral cortex (Cx) and splenium of corpus callosum (scc) are shown in the Fig.; scale bars: 100 μm. Higher magnifications are shown in panel C (bottom); scale bars: 20 μm

It was recently shown that the selective ablation of *Npc1* expression in oligodendrocytes results in defective myelin formation in the forebrain and corpus callosum of PN16 mice [39], indicating that these cells need exogenous cholesterol uptake at least during early postnatal life. This finding is in agreement with previous observations showing the expression of low-/very low-density lipoprotein receptors by oligodendrocytes [40] and the dependence on glia-derived cholesterol of Npc1-deficient brains [41, 42]. Moreover, dysmyelination and myelin loss were previously reported in prefrontal cortex, corpus callosum and hippocampus of *Npc1*^*−/−*^ mice [43] and found to be associated with defective genetic control of oligodendrocyte differentiation [44]. To investigate whether/how Npc1-deficiency also affected myelin formation during cerebellum development, we determined the expression of myelin basic protein (MBP), a well-established marker of mature myelin [45] in PN11 and PN15 cerebella of *Npc1*^*nmf164*^ and *wt* mice. As shown by Western blot analysis (Fig. 8a), the level of MBP isoforms was significantly reduced with respect to *wt* at either PN11 (main effect of genotype: 17.2 kDa: t_6_ = 4.21, *p* = 0.006; 18.5 kDa: t_6_ = 4.38, *p* = 0.005; 21.5 kDa: t_6_ = 4.03, *p* = 0.007) and PN15 (main effect of genotype: 17.2 kDa: t_6_ = 4.76, *p* = 0.003; 18.5 kDa: t_6_ = 3.51, *p* = 0.01; 21.5 kDa: t_6_ = 21.86, *p* = 0.000001). Various MBP isoforms are generated by alternative splicing and exert specific functions in different intracellular compartments. Namely, the 17.2 and 21.5 kDa isoforms are highly expressed in the cell body and nucleus of developing oligodendrocytes, playing a regulatory role in the genetic program of oligodendrocyte differentiation [46]. In contrast, the 18.5 kDa isoform localizes at the plasma membrane and actively participates in membrane compaction typical of mature myelin [47].

Immunohistochemistry of PN15 cerebellar sections fully confirmed the impairment of myelin formation in *Npc1*^*nmf164*^ mice, showing a significant reduction of MBP immunostaining at the level of PC axons and white matter (Fig. 8b). To further characterize this defect, similar analyses were also performed on cerebral cortex and corpus callosum, i.e. brain areas in which individual oligodendrocytes are more easily detected (Fig. 8c). Based on these analyses, the dysmyelination of *Npc1*^*nmf164*^ mice appeared to be associated with poor oligodendrocyte differentiation, as indicated by the reduced length of the processes that typically radiate from oligodendrocyte soma. Accordingly, the MBP immunostaining of cerebral cortex and corpus callosum was strongly reduced, in agreement with previous observations [43].

### CD treatment partially rescues the abnormal development of glial and neuronal cells in *Npc1*^*nmf164*^ mice

**Fig. 9:**
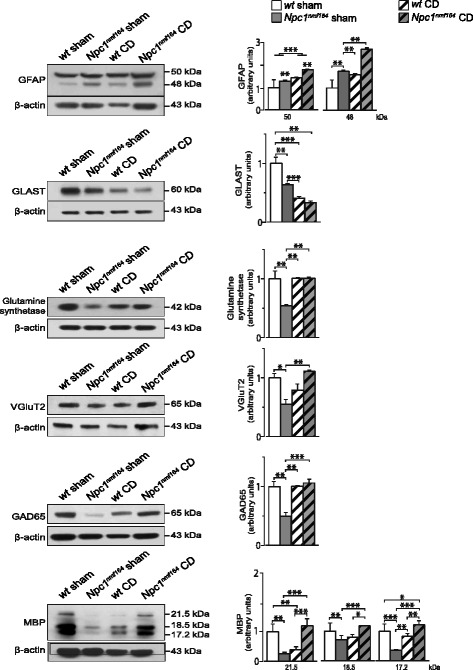
CD administration partly rescues morpho/functional markers of glial and neuronal cells. Representative western blot analyses of total protein preparations obtained from PN15 *wt* and *Npc1* ^*nmf164*^ mice, either sham- or CD-treated, and probed with specific antibodies. Histograms indicate the abundance (mean ± SEM) of each protein determined by densitometry of protein bands of at least 3 independent experiments taking β-actin as internal reference. * *p* ≤ 0.01, ** *p* < 0.001, *** *p* < 0.0001

A single CD administration to PN7 *Npc1*^*−/−*^ mouse pups was shown to rescue cholesterol defects, extend life span [17] and restore normal patterns of cerebellar granule proliferation [11]. Therefore, to determine whether early postnatal CD treatment re-established normal patterns of glial and neuronal morphological/functional markers, we performed Western blot analyses of protein extracts obtained from cerebella of PN15 *wt* and *Npc1*^*nmf164*^ mice, either sham- or CD-treated as previously described [20]. Results of this survey (Fig. 9) can be summarized as follows: first, *wt* and *Npc1*^*nmf164*^ sham-treated mice displayed differences in protein levels similar to those observed between *wt* and *Npc1*^*nmf164*^ naive mice (compare data of Figs. 4, 5, 6, 7, and 8 to those of Fig. 9), ruling out the possibility that the injection *per se* altered protein expression; second, CD administration somehow influenced the expression of GFAP, GLAST and MBP of *wt* mice, while having no apparent effect on Glutamine synthetase, VGluT2 and GAD65 (see Table 4 for two-way ANOVA analyses); third, CD administration to *Npc1*^*nmf164*^ mice fully rescued the decrease of Glutamine synthetase, VGluT2, GAD65 and MBP, restoring the protein levels to those of either sham or CD-treated *wt* mouse cerebella (Table 4). By contrast, CD administration did not rescue GFAP and GLAST expression levels in *Npc1*^*nmf164*^ mice. Indeed, GFAP and GLAST protein levels of either sham- or CD-treated *Npc1*^*nmf164*^ mice were significantly higher (GFAP) and lower (GLAST), respectively, of those of *wt* mice. Immunohistochemical assays confirmed results obtained by Western blot analyses, showing that CD treatment re-wired the expression of VGluT2 (paradigmatic of neuronal functional marker), but not GFAP (paradigmatic of a glial morpho-functional marker) (Additional file 5: Figure S4, Additional file 6: Figure S5).

**Table 4:** Statistical analysis outputs on Western blot assays of *wt* and *Npc1* ^*nmf164*^ littermates either sham- or CD-treated

		Genotype^a^	Treatment^b^	Genotype x treatment
GFAP	50 kDa	*F*_(1,12)_ = 29.35, *p* = 0.0001	*F*_(1,12)_ = 52.17, *p* < 0.0001	*F*_(1,12)_ = 0.70, *p* = 0.42
	48 kDa	*F*_(1,12)_ = 199.21, *p* < 0.0001	*F*_(1,12)_ = 152.93, *p* < 0.0001	*F*_(1,12)_ = 8.78, *p* = 0.01
GLAST		*F*_(1,12)_ = 22.77, *p* = 0.0005	*F*_(1,12)_ = 95.83, *p* < 0.0001	*F*_(1,12)_ = 9.63, *p* = 0.01
Glutamine Synthetase		*F*_(1,12)_ = 12.85, *p* = 0.004	*F*_(1,12)_ = 12.51, *p =* 0.004	*F*_(1,12)_ = 11.48, *p* = 0.005
VGluT2		*F*_(1,12)_ = 0.83, *p* = 0.38	*F*_(1,12)_ = 5.47, *p =* 0.04	*F*_(1,12)_ = 26.21, *p* = 0.0003
GAD65		*F*_(1,12)_ = 13.53, *p* = 0.003	*F*_(1,12)_ = 22.02, *p =* 0.0005	*F*_(1,12)_ = 21.89, *p* = 0.0005
MBP	21.5 kDa	*F*_(1,12)_ = 0.14, *p* = 0.71	*F*_(1,12)_ = 3.37, *p =* 0.09	*F*_(1,12)_ = 74.51, *p* < 0.0001
	18.5 kDa	*F*_(1,12)_ = 0.04, *p* = 0.84	*F*_(1,12)_ = 5.60, *p =* 0.04	*F*_(1,12)_ = 24.13, *p* = 0.0004
	17.2 kDa	*F*_(1,12)_ = 1.55, *p* = 0.24	*F*_(1,12)_ = 47.36, *p* < 0.0001	*F*_(1,12)_ = 92.86, *p* < 0.0001

^a^differences were analyzed by two-way ANOVAs

^b^ *wt* and *Npc1* ^*nmf164*^ littermates received subcutaneous injections of plain PBS or CD, according to the schedule of Fig. 1

## Discussion

We have shown that *Npc1*^*nmf164*^ mice acquire fine motor coordination and balance as well as complex abilities depending on cerebellar maturation [48, 49] with a significant delay compared to *wt* littermates, in spite of their normal physical and postural development. An overall disturbance of cerebellar morphogenesis underscores this phenotype, emphasizing the relevance of exogenous cholesterol uptake and Npc1-mediated intracellular trafficking for proper cerebellar development. The presence of a developmental delay instead of a more severe deficit of these abilities is likely explained by the greater plasticity of developing cerebellum and/or the availability of cholesterol of neuronal origin before the full shift to astrocyte-derived cholesterol, which compensates the deficit of Npc1 function.

The normal appearance of sensorimotor reflexes and locomotion development of *Npc1*^*nmf164*^ pups within the first two postnatal weeks indicate that vestibular, tactile, and proprioceptive systems; descending motor pathways; and brain stem-spinal networks [30, 49] are not apparently affected by Npc1-deficiency. Conversely, the domain of complex motor abilities is damaged by Npc1-deficiency because they are also prematurely lost in the adulthood. In fact, motor coordination and balance are more severely impaired than grip capacity and muscle strength as early as at PN30 and these motor defects thereafter translate to severe ataxia, because of the massive PC degeneration [15, 24].

The acquisition of complex motor abilities depends on proper sequencing and coordination of motor outputs. These are prominent properties of cerebellar circuitry [48], consisting of several functional modules that allow the real-time control of movements and the long-term changes underling motor learning, by finely regulated signal generation and flow that ultimately converge on PCs. The inhibitory activity of PCs is dynamically orchestrated at the level of both dendritic shafts and cell body by a number of excitatory and inhibitory neurons, while PCs in turn modulate the excitability of deep cerebellar nuclei. Therefore, an altered pattern of synaptic inputs to PCs may affect the timing of their firing and finally result in behavior abnormalities. For example, it was recently demonstrated that an altered GN development results in impaired motor coordination [50, 51]. Similar features were also reported as a consequence of a defective development of BG processes [52–54], likely because correct BG development is crucial for cerebellar cytoarchitecture and function [52]. Conversely, a precocious BG and PC maturation is associated with an earlier acquisition of motor abilities in young and improved motor learning and coordination in adult mice [55]. In light of these findings, it is possible that the developmental delay in the acquisition of complex motor skills we have observed in *Npc1*^*nmf164*^ pups results from a derangement of synaptic inputs to PCs.

Indeed, we found several developmental anomalies that impinge on the functionality of PCs, suggesting the possibility that the selective vulnerability of these cells represents the final outcome of a number of developmental defects in glial and neuronal cells forming the ordered pattern of cell-to-cell interaction and synaptic connectivity of cerebellar cortex [56]. For instance, the abnormal BG differentiation (thicker radial shafts and a less elaborate reticular pattern of lateral processes) we observe in PN15 *Npc1*^*nmf164*^ mice may be particularly relevant. In fact, BG processes organize the compartmentalization pattern of synaptic inputs that reach PCs [36], playing a prominent role in the differential guidance and targeting of basket and stellate cell axons [37]. In addition, BG processes finely regulate cerebellar synaptic activity [57] by almost completely enwrapping the synapses that parallel and climbing fibers establish with PCs [14, 36].

The glutamate transporter GLAST finely regulates PC firing at BG perisynaptic processes by preventing glutamate spillover between adjacent PCs [35] and maintaining the one-to-one functional relationship between climbing fibers and PCs that is crucial for cerebellar control of motor function [54, 58, 59]. Because the glutamate recovered by GLAST is metabolized to glutamine by Glutamine synthetase [60], the reduced expression of GLAST and Glutamine synthetase in *Npc1*^*nmf164*^ mice is in line with the proposal that GLAST is a limiting factor in glutamate synthesis [61]. Also, GFAP plays a key role in astrocyte-neuron interactions, by modulating the trafficking and function of astrocytic and neuronal glutamate transporters, as well as glutamine production [62]. All together these findings suggest that the abnormal morphological differentiation of BG affects the functional specialization of their processes, as also indicated by the reduced expression of GLAST and Glutamine synthetase. In addition to BG, a decrease of GLAST and Glutamine synthetase expression is likely to occur in astrocytes, the functional impairment of which is indicated by astrocytosis typically displayed by *Npc1*^*nmf164*^ cerebella. In this regard it is worth noting that, in Npc1-deficient mice, astrocytosis is consistently accompanied by microglia activation [15, 63, 64] and the down regulation of GLAST has been found to correlate with the release of inflammatory cytokines by activated microglia [65].

Npc1 is also abundant in the recycling endosomes of presynaptic terminals. In fact, Npc1-deficiency results in morphological, biochemical and functional modification of both excitatory and inhibitory presynaptic terminals and synaptic vesicle turnover [66]. This may explain the reduction of both excitatory and inhibitory inputs received by PCs, as indicated by the significant reduction of VGluT2 and GAD65 puncta. This imbalance of synaptic inputs associated with Npc1-deficiency is in agreement with previous findings showing a decrease of synaptic inputs to PCs in co-cultures of Npc1-deficient neurons and glial cells [67]. The lower number of GNs that are generated in the cerebellum of *Npc1*^*nmf164*^ mice likely contributes to the reduction in glutamatergic inputs, as indicated by our finding that VGluT2 puncta reduction is prominent in the outer molecular layer, which is mostly made of GN axons. On the other hand, the GN reduction may also impinge on the full differentiation of basket/stellate interneurons, which, among other intrinsic genetic programs and extracellular cues, depends on connectivity with GN axons [68, 69]. Along the same line, the lower number of GNs may also be responsible for the abnormal differentiation of BG processes in *Npc1*^*nmf164*^ mice, because the glutamate released by parallel fibers modulates the degree of BG perisynaptic envelopment acting through calcium-permeable AMPA receptors [57].

Defective oligodendrocyte maturation likely underlines the overall reduction in MBP we have observed in *Npc1*^*nmf164*^ cerebellum, in line with previous studies showing dysmyelination in both NPC patients and *Npc1*^−/−^ mice [43, 70]. Moreover, the decreased MBP expression not only affected the 18.5 kDa (specific to mature oligodendrocytes), but also the 17.5 kDa and 21.5 kDa isoforms (specific to developing oligodendrocytes), indicating that oligodendrocyte differentiation *per se* is also impaired in Npc1-deficient mice. Although Npc1 deletion in neurons triggers the block of oligodendrocyte maturation and thus leads to a subsequent failure of myelin formation [39], the exogenous cholesterol uptake by oligodendrocytes coupled to Npc1-mediated intracellular trafficking is also relevant for the formation of myelin sheaths [40, 44]. Accordingly, oligodendrocyte ablation during the first postnatal weeks gives rise to ataxia and motor deficits in the mouse [39, 71]. By showing that a significant myelin reduction is prominent at the level of PC axons, our results further corroborate the convergence of various Npc1 deficiency-dependent abnormalities on PC functionality.

Present findings also demonstrate that early postnatal CD treatment effectively re-wires developmental trajectories, by partly rescuing the defective cerebellar morphogenesis and thus explaining the well-established beneficial effect a single CD administration to PN7 *Npc1*^*−/−*^ mice has in rescuing lysosomal cholesterol accumulation and slowing down the appearance of ataxic symptoms [17–19, 72, 73]. In fact, this treatment is particularly timely because cerebellar morphogenesis maximizes the need for cholesterol, making the exogenous LDL-uptaken cholesterol a rate-limiting factor for neurons [74, 75]. Noteworthy, CD administration didn’t rescue either GFAP hyper- or GLAST hypo-expression of *Npc1*^*nmf164*^ mice, although it fully rescued Glutamine synthetase levels in these mice. Because Glutamine synthetase is mainly expressed by astrocytes, this observation rules out the possibility that astrocytes are not influenced by CD, making this issue worthy of further investigation, also in light of the ability of CD administration to influence GFAP and GLAST expression of *wt* mice.

## Conclusions

In conclusion, we correlate the delay of complex motor skills acquisition by *Npc1*^*nmf164*^ mice to a number of glial cell differentiation anomalies and derangement of synaptic input to PCs. We believe that these findings are relevant because: i) delineate a novel perspective to explain the selective Purkinje cell vulnerability in NPC1 mouse models and patients; and, ii) emphasize the need of early diagnosis to secure the best treatment efficacy in patients.

## Additional files

Additional file 1: Supplementary materials and methods. (DOCX 127 kb)

Additional file 2: Figure S1. *Npc1* ^*nmf164*^ mice display a reduced density of GNs in the external granule layer (EGL), which is due to reduced proliferation of GN precursors. **A** Representative sections are shown in the figure. Higher magnification fields of EGL base or crown of lobules II and X on the right of low magnification fields show that the EGL of PN15 *Npc1* ^*nmf164*^ mice is thinner than that of age-matched *wt* mice. Scale bar indicate 250 μm (panels) and 50 μm (insets). **B** Histograms represent GN densities (mean ± SEM of all sections examined; *N* = 4 mice/genotype; 3–4 sections/mouse) determined in 100 μm^2^ regions of the crowns of *wt* and *Npc1* ^*nmf164*^ mice anterior (I-V) and posterior (VI-X) lobules. **C** A representative field showing BrdU-positive cells (red) of fissure between lobules II and III of PN13 *wt* and *Npc1* ^*nmf164*^ mice. Scale bar indicates 50 μm. **D** Histograms represent the number of BrdU-positive cells (mean ± SEM; 4 mice/genotype; 3–4 sections/mouse) determined in 100 μm^2^ regions corresponding to the bases and crowns of PN13 and PN15 *wt* and *Npc1* ^*nmf164*^ mice anterior (I–V) and posterior (VI–X) lobules. Asterisks indicate statistically significant differences (unpaired two-tailed Student’s *t* test, ** *p* < 0.001; *** *p* < 0.0001). (PDF 1513 kb)

Additional file 3: Figure S2. **A** Western blot analysis of GFAP protein expression in cerebella of PN11 *wt* and *Npc1* ^*nmf164*^ mice. **B** Histograms indicate the abundance (mean ± SEM) of each isoform determined by densitometry of protein bands obtained in at least 3 independent experiments taking β-actin as internal reference. (PDF 95 kb)

Additional file 4: Figure S3. The cerebellar cortex of PN15 *wt* and *Npc1* ^*nmf164*^ mice diplays similar densities of Bergmann glia, Purkinje cells and basket/stellate interneurons. The number of Bergmann glia, PCs and basket/stellate interneurons was determined in cerebellar sections of PN15 *wt* and *Npc1* ^*nmf164*^ mice stained with hematoxylin/eosin Y (right panel; asterisks: migrating GNs; arrows: basket/stellate interneurons; arrowheads: Bergmann glia) or processed for immunostaining with anti-parvalbumin antibody (left panel) to identify GABA-ergic neurons/interneurons. Scale bar: 50 μm. Histograms represent cell densities (mean ± SEM of all sections examined; *N* = 3 mice/genotype; 3–4 sections/mouse) determined in 0.04 mm^2^ regions randomly selected in each microscopic field of anterior (I-V) and posterior (VI-X) lobules of *wt* and *Npc1* ^*nmf164*^ mouse cerebella, stained with hematoxylin/eosin Y (right) or anti-parvalbumin antibody (left). Since any significant difference was found between counts of anterior and posterior lobules, values were averaged. Comparisons were performed by unpaired two-tailed Student’s *t* test. (PDF 3404 kb)

Additional file 5: Figure S4. CD treatment fully rescued VGluT2 puncta reduction of *Npc1* ^*nmf164*^ mice. **A** Immunostaining with antibodies directed to VGluT2 (brown) of PN15 *wt* and *Npc1* ^*nmf164*^, either sham- or CD-treated mouse cerebella. Representative fields of parasagittal sections are shown in the figure. Upper panels, arrows indicate VGluT2-positive synapses of internal granule layer glomeruli; scale bars: 20 μm. Bottom panels, higher magnifications of selected areas. Arrowheads indicate VGluT2 positive puncta; scale bars: 5 μm. **B** Histograms indicate VGluT2-positive puncta densities in the outer and inner molecular layers (mean ± SEM of all sections examined; *N* = 4 mice/genotype/treatment; 3–4 sections/mouse) of *wt* and *Npc1* ^*nmf164*^ mice, either sham- or CD-treated. Asterisks indicate statistically significant differences (two-way ANOVA, * *p* < 0.01). (PDF 1101 kb)

Additional file 6: Figure S5. CD treatment does not rescue defective BG morphology and astrocyte activation. Immunostaining with antibodies directed to GFAP (brown) of PN15 *wt* and *Npc1* ^*nmf164*^, either sham- or CD-treated mouse cerebella. Note that CD-treated *wt* mice display enlarged radial shaft and hypertrophic astrocytes similar to those of *Npc1* ^*nmf164*^. Representative fields of parasagittal sections are shown; scale bar indicate 50 μm. Higher magnification fields are shown on the right; scale bars: 25 μm. ML: Molecular Layer; PCL: Purkinje Cell Layer; IGL: Internal Granular Layer. (PDF 1170 kb)
