# Supplementary material for: Developmental delay in motor skill acquisition in Niemann-Pick C1 mice reveals abnormal cerebellar morphogenesis
Source: Acta Neuropathol Commun. 2016 Sep 1;4(1):94. doi: 10.1186/s40478-016-0370-z (PMC5009663; doi:10.1186/s40478-016-0370-z)
Supplement: Supplementary file 1 — Supplementary materials and methods. (DOCX 127 kb) [file 40478_2016_370_MOESM1_ESM.docx]

**Additional File 1 (file format: Microsoft word DOCX)**

**Supplementary Materials and Methods**

*Histology and immunohistochemostry*

To determine the number of GNs, brains were dissected from PN15 *wt* and *Npc1^nmf164^*, fixed in 4% PFA, dehydrated, embedded in Paraplast Tissue Embedding Medium (Leica Biosystem, Milan, Italy) and serially sectioned (slice thickness 8 μm). Sagittal sections were then stained with Hoechst (Hoechst-33258, Invitrogen, Milan, Italy) as previously described [11]. To assess GN proliferation, brains were dissected from PN13 and PN15 *wt* and *Npc1^nmf164^* mice that had received a single BrdU injection 20 hr before and processed as previously described [11]. Briefly, epitopes were unmasked by heating sections 2 × 5 min in 10 mM sodium citrate, pH 6.0, in a microwave oven and tissue permeabilization was achieved by incubation in trypsin solution (0.05% trypsin, 0.1% CaCl_2_ in water) for 15 min at room temperature (RT) and then in 2 M HCl for 30 min. Histological sections were then incubated overnight at 4 °C with a monoclonal anti-BrdU antibody (Immunological Science, Rome, Italy; 1:100 dilution in PBS supplemented with 0.5% Tween 20), and then for 45 min with an anti-mouse IgG antibody (Alexa Fluor-555 InVitrogen, Milan, Italy; 1:1500 final dilution). After counterstaining with Hoechst, sections were mounted with Prolong Gold Antifade Reagent and analyzed with an epifluorescence Zeiss microscope. For quantification of BrdU-immunopositive cells, regions of interest were randomly selected from bases and crowns of the anterior (I-V) and posterior (VI-X) lobules. The number of BrdU-positive cells in each region was determined as fraction of the total cell number. Hematoxylin/eosin Y staining was performed as previously described [20]. For parvalbumin immunostaining, paraffin sections of PN15 *wt* and *Npc1^nmf164^* mice (obtained as described under the Materials and Methods section) were subjected to a blocking/permeabilization step consisting in a 30 min incubation in PBS supplemented with 5% normal goat serum, 0.3% Triton X-100. Sections were then incubated with a monoclonal anti-parvalbumin antibody (Sigma Aldrich, Milan, Italy; 1:700 dilution in PBS supplemented with 3% normal goat serum) overnight at 4°C, washed 3-4 times with PBS and stained using the mouse Vectastain Elite ABC kit and the DAB peroxidase substrate kit (Vector Laboratories Inc., Burlingame, CA, USA) according to the protocol supplied by the manufacturer.
