# Supplementary figures and images for: Developmental delay in motor skill acquisition in Niemann-Pick C1 mice reveals abnormal cerebellar morphogenesis
Source: Acta Neuropathol Commun. 2016 Sep 1;4(1):94. doi: 10.1186/s40478-016-0370-z (PMC5009663; doi:10.1186/s40478-016-0370-z)

Figure S1

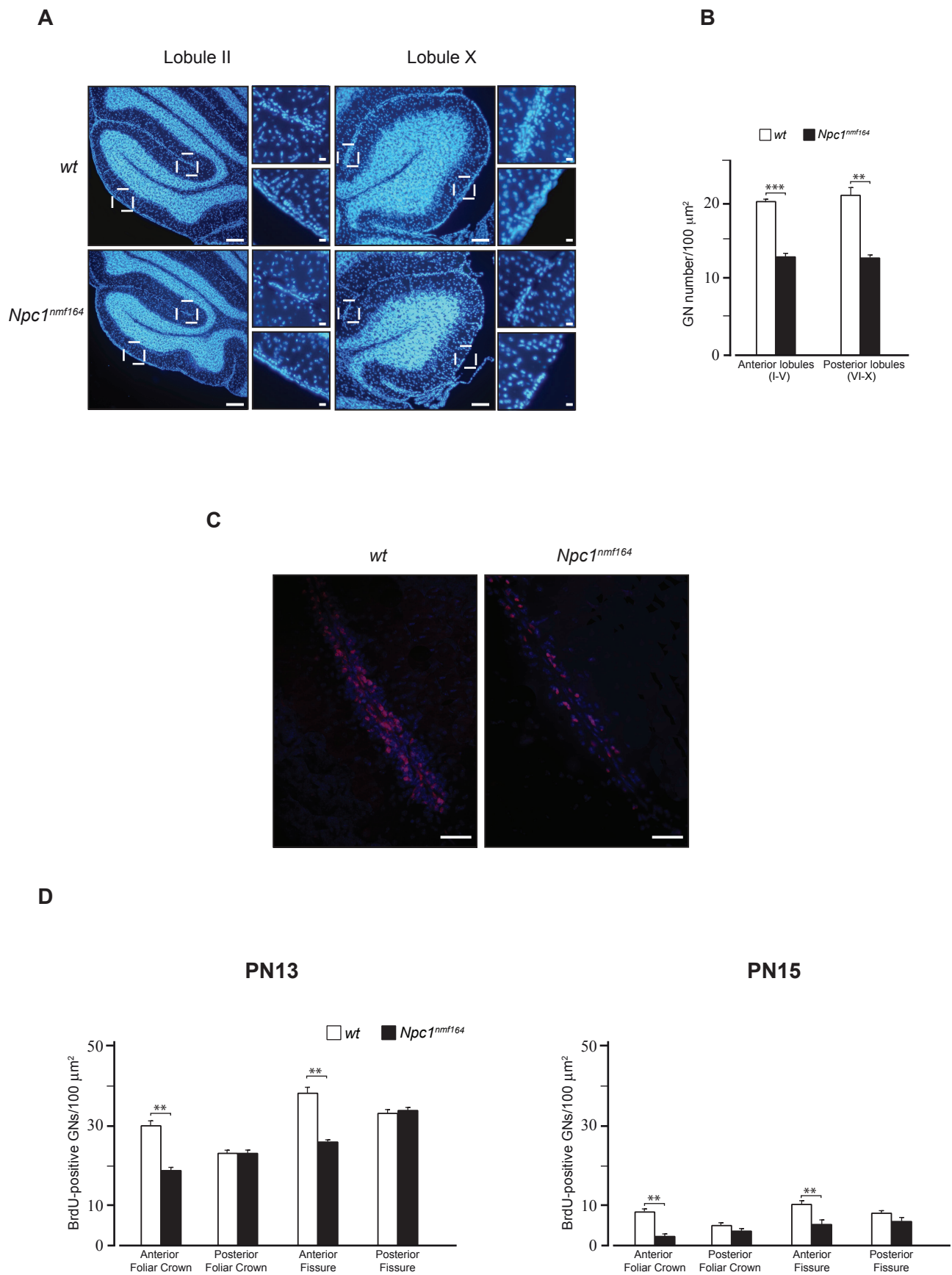

Supplement: Supplementary file 2 — Npc1 nmf164 mice display a reduced density of GNs in the external granule layer (EGL), which is due to reduced proliferation of GN precursors. A Representative sections are shown in the figure. Higher magnification fields of EGL base or crown of lobules II and X on the right of low magnification fields show that the EGL of PN15 Npc1 nmf164 mice is thinner than that of age-matched wt mice. Scale bar indicate 250 μm (panels) and 50 μm (insets). B Histograms represent GN densities (mean ± SEM of all sections examined; N = 4 mice/genotype; 3–4 sections/mouse) determined in 100 μm2 regions of the crowns of wt and Npc1 nmf164 mice anterior (I-V) and posterior (VI-X) lobules. C A representative field showing BrdU-positive cells (red) of fissure between lobules II and III of PN13 wt and Npc1 nmf164 mice. Scale bar indicates 50 μm. D Histograms represent the number of BrdU-positive cells (mean ± SEM; 4 mice/genotype; 3–4 sections/mouse) determined in 100 μm2 regions corresponding to the bases and crowns of PN13 and PN15 wt and Npc1 nmf164 mice anterior (I–V) and posterior (VI–X) lobules. Asterisks indicate statistically significant differences (unpaired two-tailed Student’s t test, ** p < 0.001; *** p < 0.0001). (PDF 1513 kb) [file 40478_2016_370_MOESM2_ESM.pdf]

Figure S2

**A**

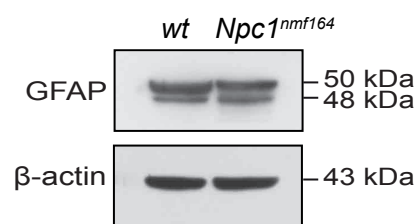

**B**

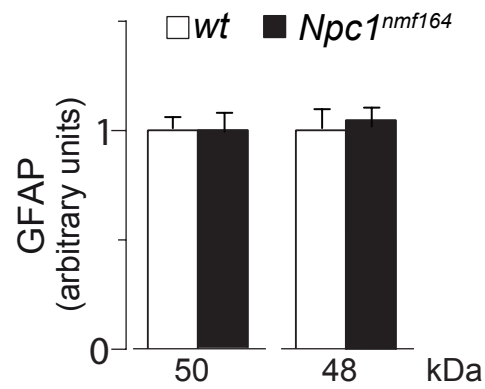

Supplement: Supplementary file 3 — A Western blot analysis of GFAP protein expression in cerebella of PN11 wt and Npc1 nmf164 mice. B Histograms indicate the abundance (mean ± SEM) of each isoform determined by densitometry of protein bands obtained in at least 3 independent experiments taking β-actin as internal reference. (PDF 95 kb) [file 40478_2016_370_MOESM3_ESM.pdf]

Figure S3

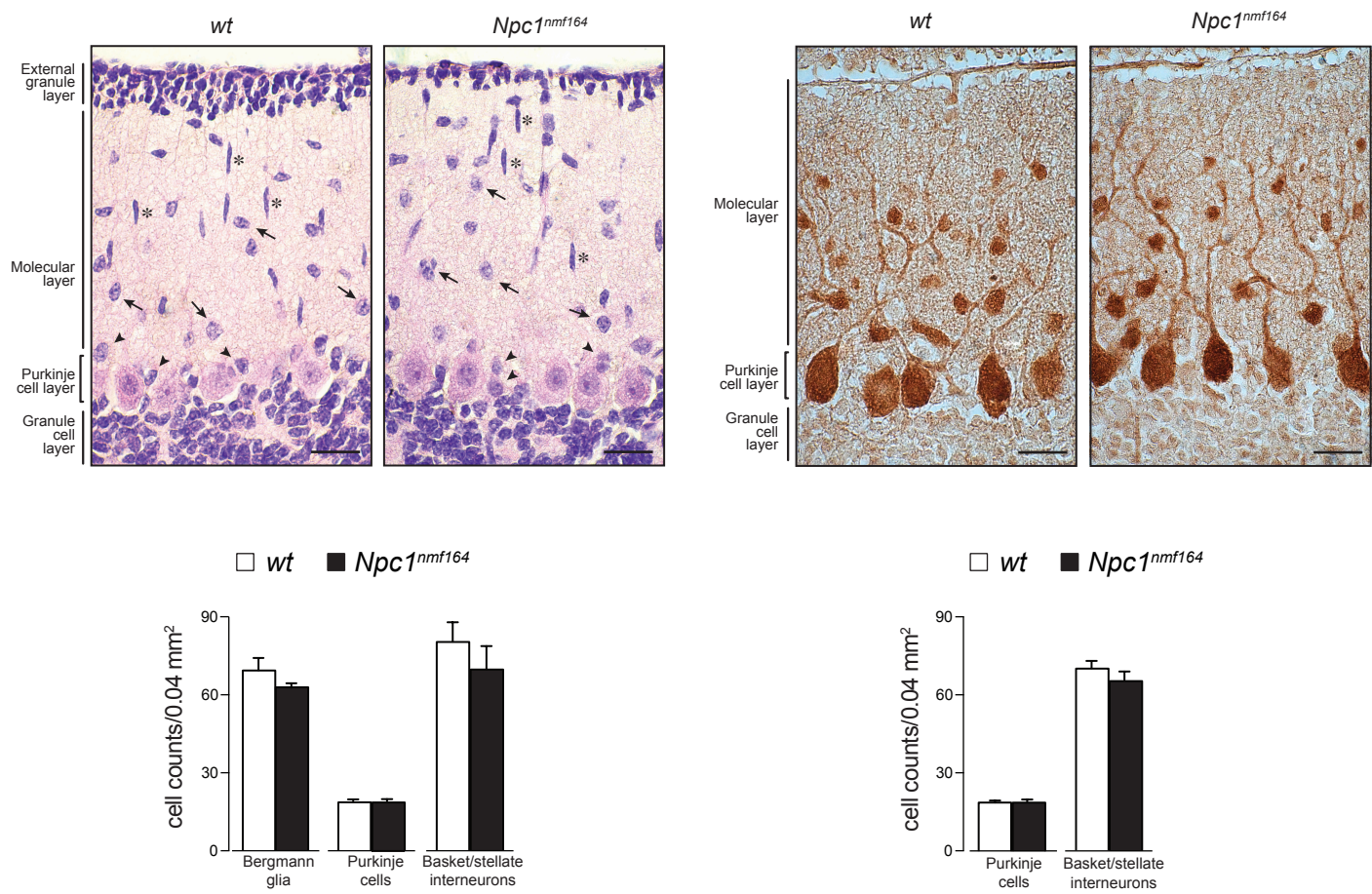

Supplement: Supplementary file 4 — The cerebellar cortex of PN15 wt and Npc1 nmf164 mice diplays similar densities of Bergmann glia, Purkinje cells and basket/stellate interneurons. The number of Bergmann glia, PCs and basket/stellate interneurons was determined in cerebellar sections of PN15 wt and Npc1 nmf164 mice stained with hematoxylin/eosin Y (right panel; asterisks: migrating GNs; arrows: basket/stellate interneurons; arrowheads: Bergmann glia) or processed for immunostaining with anti-parvalbumin antibody (left panel) to identify GABA-ergic neurons/interneurons. Scale bar: 50 μm. Histograms represent cell densities (mean ± SEM of all sections examined; N = 3 mice/genotype; 3–4 sections/mouse) determined in 0.04 mm2 regions randomly selected in each microscopic field of anterior (I-V) and posterior (VI-X) lobules of wt and Npc1 nmf164 mouse cerebella, stained with hematoxylin/eosin Y (right) or anti-parvalbumin antibody (left). Since any significant difference was found between counts of anterior and posterior lobules, values were averaged. Comparisons were performed by unpaired two-tailed Student’s t test. (PDF 3404 kb) [file 40478_2016_370_MOESM4_ESM.pdf]

Figure S4

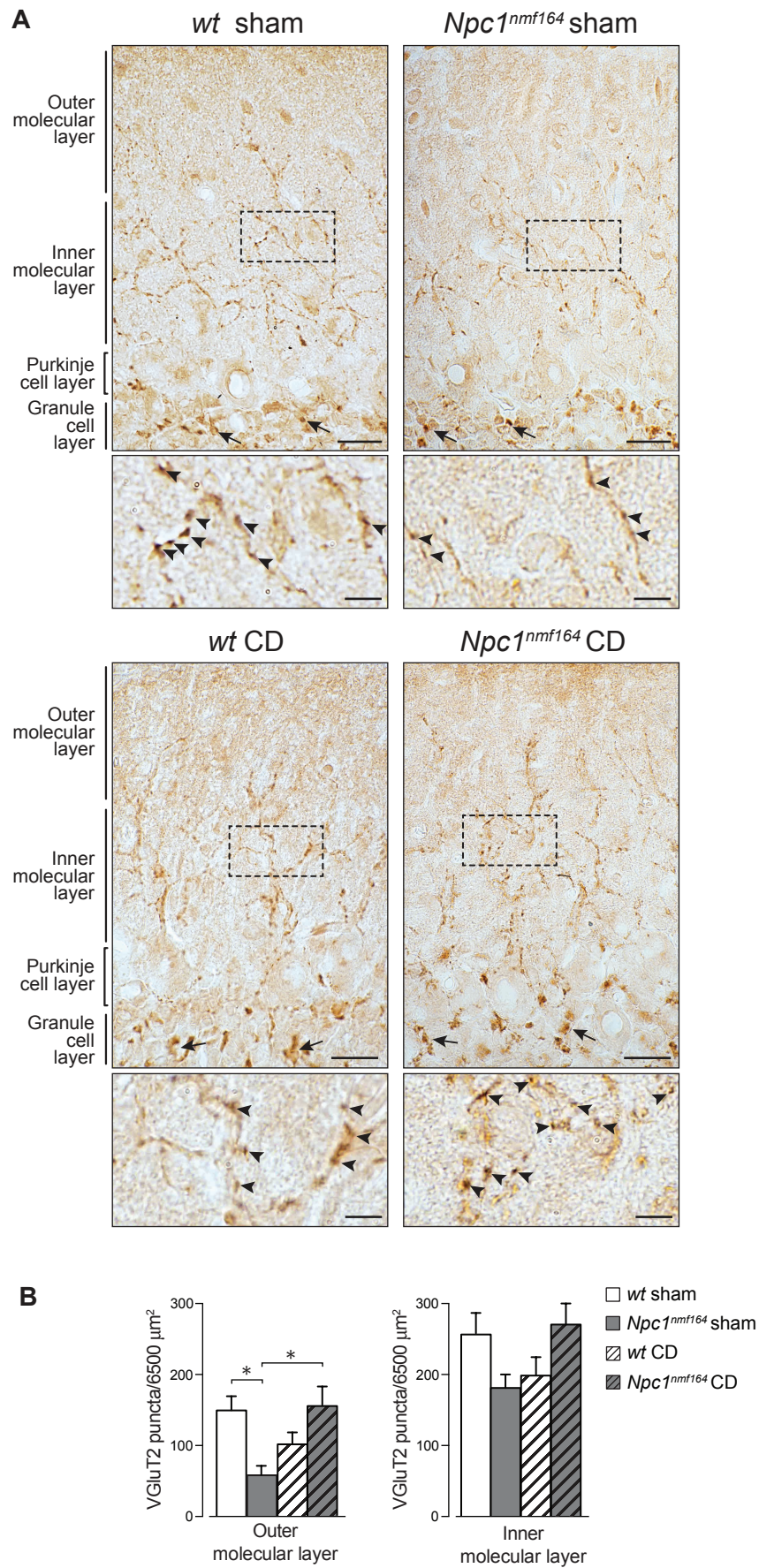

Supplement: Supplementary file 5 — CD treatment fully rescued VGluT2 puncta reduction of Npc1 nmf164 mice. A Immunostaining with antibodies directed to VGluT2 (brown) of PN15 wt and Npc1 nmf164, either sham- or CD-treated mouse cerebella. Representative fields of parasagittal sections are shown in the figure. Upper panels, arrows indicate VGluT2-positive synapses of internal granule layer glomeruli; scale bars: 20 μm. Bottom panels, higher magnifications of selected areas. Arrowheads indicate VGluT2 positive puncta; scale bars: 5 μm. B Histograms indicate VGluT2-positive puncta densities in the outer and inner molecular layers (mean ± SEM of all sections examined; N = 4 mice/genotype/treatment; 3–4 sections/mouse) of wt and Npc1 nmf164 mice, either sham- or CD-treated. Asterisks indicate statistically significant differences (two-way ANOVA, * p < 0.01). (PDF 1101 kb) [file 40478_2016_370_MOESM5_ESM.pdf]

Figure S5

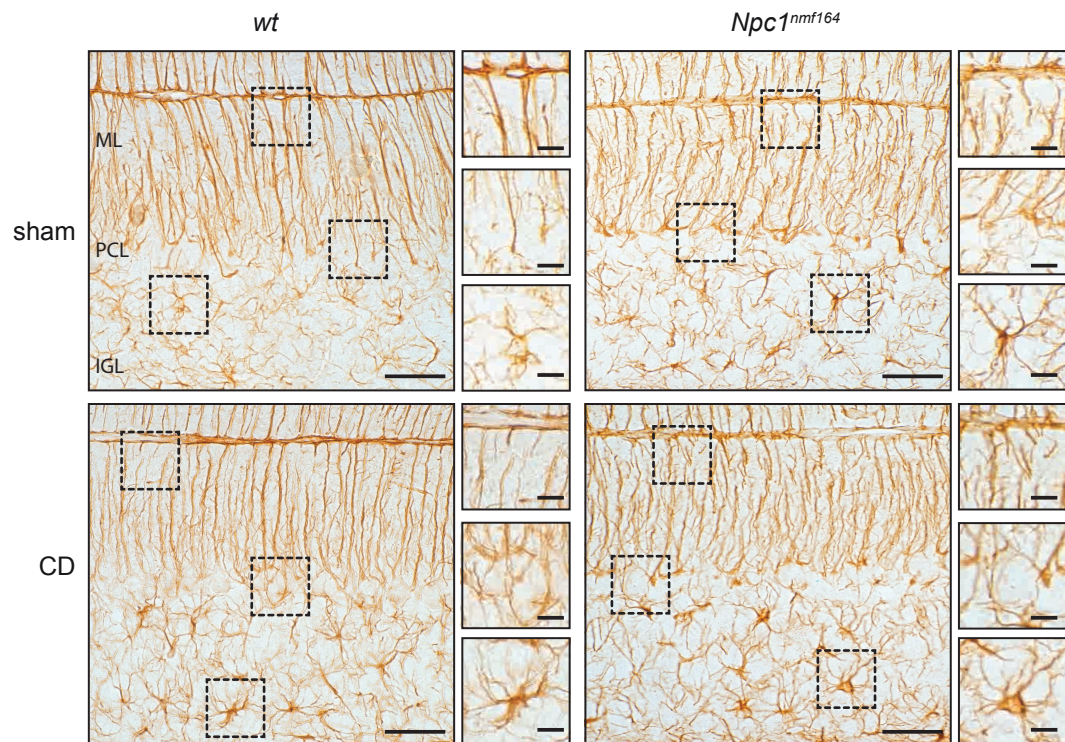

Supplement: Supplementary file 6 — CD treatment does not rescue defective BG morphology and astrocyte activation. Immunostaining with antibodies directed to GFAP (brown) of PN15 wt and Npc1 nmf164, either sham- or CD-treated mouse cerebella. Note that CD-treated wt mice display enlarged radial shaft and hypertrophic astrocytes similar to those of Npc1 nmf164. Representative fields of parasagittal sections are shown; scale bar indicate 50 μm. Higher magnification fields are shown on the right; scale bars: 25 μm. ML: Molecular Layer; PCL: Purkinje Cell Layer; IGL: Internal Granular Layer. (PDF 1170 kb) [file 40478_2016_370_MOESM6_ESM.pdf]
